# Developmental Changes in Pyramidal Cell Morphology in Multiple Visual Cortical Areas Using Cluster Analysis

**DOI:** 10.3389/fncom.2021.667696

**Published:** 2021-05-31

**Authors:** Reem Khalil, Ahmad Farhat, Paweł Dłotko

**Affiliations:** ^1^Biology, Chemistry, and Environmental Sciences Department, American University of Sharjah, Sharjah, United Arab Emirates; ^2^Dioscuri Centre in Topological Data Analysis, Mathematical Institute, Polish Academy of Sciences, Warsaw, Poland

**Keywords:** monkey, refinement, PCA, development, V1, clustering, mammalian brain, cerebral cortex

## Abstract

Neuronal morphology is characterized by salient features such as complex axonal and dendritic arbors. In the mammalian brain, variations in dendritic morphology among cell classes, brain regions, and animal species are thought to underlie known differences in neuronal function. In this work, we obtained a large dataset from http://neuromorpho.org/ comprising layer III pyramidal cells in different cortical areas of the ventral visual pathway (V1, V2, V4, TEO, and TE) of the macaque monkey at different developmental stages. We performed an in depth quantitative analysis of pyramidal cell morphology throughout development in an effort to determine which aspects mature early in development and which features require a protracted period of maturation. We were also interested in establishing if developmental changes in morphological features occur simultaneously or hierarchically in multiple visual cortical areas. We addressed these questions by performing principal component analysis (PCA) and hierarchical clustering analysis on relevant morphological features. Our analysis indicates that the maturation of pyramidal cell morphology is largely based on early development of topological features in most visual cortical areas. Moreover, the maturation of pyramidal cell morphology in V1, V2, V4, TEO, and TE is characterized by unique developmental trajectories.

## Introduction

The dendritic morphologies of neurons can vary markedly across cortical areas. Structural differences among neuronal cell types are believed to result in functional differences (Mainen and Sejnowski, 1996; Vetter et al., 2001; Krichmar et al., 2002; Schaefer et al., 2003; Brette et al., 2007). Much effort has focused on studying pyramidal cell morphology as this cell class is the most abundant type in the cerebral cortex (70–80% of the total neuronal population) (DeFelipe and Fariñas, 1992), and can vary considerably among cortical regions, layers, and species (Chen et al., 2009; Elston and Manger, 2014; Luebke et al., 2015; Mohan et al., 2015; Fernandez-Gonzalez et al., 2017; Hasse et al., 2019, for reviews see, Elston, 2002, 2007; Jacobs and Scheibel, 2002; Luebke, 2017). A number of studies have revealed significant regional differences in pyramidal cell morphology in the adult monkey visual cortex (Elston and Rosa, 1997, 1998, 2000; Elston et al., 1999a,b). Specifically, pyramidal cell size, dendritic complexity, and spine density increase from primary visual cortex (V1) to higher order visual areas (Elston, 2003). Pronounced variations in pyramidal cell morphology are thought to generate functional specificity within an area which is important for visual processing. For example, highly branched and more spinous dendrites of pyramidal neurons in higher order visual areas such as inferotemporal cortex (IT) are likely to integrate more inputs and sample a greater portion of the visual map than cells in V1 (Elston and Rosa, 1997).

An important developmental question is whether cortical circuits mature simultaneously or with different time scales. Currently, there are two theories that characterize the sequence of cortical development and maturation. The classical view proposes a hierarchical sequence of development whereby primary visual cortex (V1) develops first followed by higher order areas. Evidence supporting this view derives largely from axonal myelination patterns (Flechsig, 1901; Girard et al., 1991; Paus et al., 2001), showing pathways to primary sensory and motor areas myelinate first, followed by higher order areas. Additional evidence to support the view of hierarchical cortical maturation stems from cellular and behavioral studies documenting the developmental sequence of various anatomical aspects of cortical maturation (Condé et al., 1996; Bourne and Rosa, 2006; Petanjek et al., 2008; Bianchi et al., 2013; Elston and Fujita, 2014). In contrast, the opposing view suggests that cortical maturation proceeds simultaneously in multiple cortical areas. Evidence from earlier work supporting concurrent maturation was largely based on synaptic counts (Rakic et al., 1986; Bourgeois et al., 1994; Guillery, 2005). However, the sequence of development and maturation of different cortical areas may not necessarily proceed in a hierarchical or simultaneous manner, but rather different aspects of development in diverse cortical areas may simply follow unique growth profiles (Elston et al., 2010). Clarifying which aspects of cortical development and maturation ensue hierarchically or simultaneously is a prerequisite for understanding neurobiological mechanisms involved in brain maturation and cognition (Chomiak and Hu, 2017).

It is well known that the refinement of visual cortical circuits is characterized by different developmental trajectories (Khalil and Levitt, 2013, 2014; Khalil et al., 2018; Danka Mohammed and Khalil, 2020). Likewise, the maturation of pyramidal cells in different visual cortical areas exhibit different rates of growth and refinement (Boothe et al., 1979; Mates and Lund, 1983a,b; Lund and Holbach, 1991; Elston et al., 2009, 2010, 2011, Elston and Fujita, 2014). For instance, layer III pyramidal cells in V1, TE, and the prefrontal cortex of the macaque monkey exhibit pronounced differences in the magnitude of spinogenesis and pruning (Elston et al., 2009). Dendritic trees of layer III pyramidal cells in primary visual cortex (V1) of the macaque monkey decrease in size from birth into adulthood (Boothe et al., 1979; Elston et al., 2010). Conversely, pyramidal cells in layer III of inferotemporal cortex (IT), grow larger dendritic trees after the peak in synaptogenesis into adulthood (Elston et al., 2011). Moreover, there is an increase in the size and complexity of the dendritic tree of layer III pyramidal cells in the anterior ventral inferotemporal cortex (IT) (Elston et al., 2011). Collectively, these studies underscore the importance of assessing neuronal morphology in different visual cortical areas throughout development. This is crucial as the refinement of morphological features is believed to underlie the maturation of neuronal physiological properties, and could be indicative of functional maturity. Therefore, the aim of this study was to investigate morphological differences of layer III pyramidal cells in cortical areas of the ventral visual pathway (V1, V2, V4, TEO, and TE) of the macaque monkey throughout development. Our objective was to determine which aspects of neuronal morphology mature early in development and which features continue to mature. Secondarily, we wished to determine if developmental changes in key morphological features occur simultaneously or hierarchically in multiple visual cortical areas. To enhance our understanding of the developmental changes that occur in the structure of pyramidal cells, we addressed these questions by leveraging publicly available data on neuronal morphology. 3D neuronal reconstructions of layer III pyramidal cells in V1, V2, V4, TEO, TE at each age were acquired from the public repository http://neuromorpho.org/ (Ascoli et al., 2007). We performed PCA on 13 morphological metrics taking into account different aspects of neuronal morphologies. Our findings reveal that topological aspects such as branch order, fractal dimension, and contraction of the dendritic tree of layer III pyramidal cells mature early in development while morphological features related to the size of the dendritic tree continue to mature. Moreover, the temporal sequence of developmental changes in key morphological features is different across visual cortical areas.

## Materials and Methods

### Data Retrieval and Morphological Features

We acquired 3D neuronal reconstructions of pyramidal cells in layer III of monkey V1, V2, V4, TEO, and TE from the public repository http://neuromorpho.org/ (Ascoli et al., 2007). This database can be accessed via a web interface and an Application Programming Interface (API) which facilitates data queries via HTTP requests. This architecture allows users to query neuronal morphometric parameters by animal species, brain regions, cell types and archive name. We queried the database for the Fujita (Elston et al., 2011; Oga et al., 2016) archive using an R client neuromorphr (Bates et al., 2020) to interact with the API. This dataset consisted of a total of 782 reconstructions that were included for analysis. Table 1 includes a summary of the sample size for each age and visual area. Analysis was carried out on five different ages: 2 days, 3 weeks, 3.5 months, 7 months, and adult (V1 only). Each neuronal reconstruction consisted of only the basal dendritic tree and therefore all analyses were carried out on the basal skirt.

**TABLE 1:** Number of layer III pyramidal cells used at different ages in all visual areas.

**Area Age**	**V1**	**V2**	**V4**	**TEO**	**TE**
2 days	25	61	37	35	22
3 weeks	41	26	26	28	37
3.5 months	28	22	31	29	27
7 months	33	43	37	39	50
Adult	105	NA	NA	NA	NA

*NA: Adult data for these visual areas was Not Available.*

All morphological data was scaled using StandardScalar (mean value = 0, standard deviation = 1) before PCA was applied. This data comprised the following 15 morphological features: **Branch length** = total arborization length, **Euclidean distance** from soma, **path distance** from soma, **branch contraction** = average contraction (the ratio between Euclidean and path length calculated on each branch), **Partition asymmetry** = topological asymmetry (average over all bifurcations of the absolute value of (n1−n2)/(n1 + n2−2), where n1 and n2 are the numbers of tips in the two subtrees), **number of neuronal stems** = number of primary dendritic branches, **number of neuronal bifurcations** = number of branchpoints, **number of neuronal branches** = number of bifurcations and terminations, **branch order** = maximum branch order (number of bifurcations from soma to tips), **remote bifurcation amplitude** = remote bifurcation angle (average over all bifurcations of the angle between the following bifurcations or tips), **Neuronal width** = adjusted width of whole arbor; **Neuronal height** = adjusted height of whole arbor; **Neuronal depth** = adjusted depth of whole arbor, **Fractal dimension of branches** = the slope of linear fit of regression line obtained from the log-log plot of Path distance vs. Euclidean distance, and **convex hull** = total dendritic field area. Convex hull was computed by using the SWC files, which contain three dimensional neuronal reconstructions in a digitized format. Each SWC file contains a digital representation of a neuron as a tree structure comprising a number of reconstruction nodes (Ascoli et al., 2007; Scorcioni et al., 2008). Each of the measurements is a single number associated with a given neuron. When the numbers are concatenated, we obtain a fifteen-dimensional feature vector of a neuron that will be used to characterize it.

### Principal Component Analysis (PCA) and Cluster Analysis

PCA is a powerful statistical technique often used to reduce the dimensionality of a dataset by using a linear combination of dimensions which explains the majority of the variance in the data. This is accomplished by transforming a high dimensional set of data points into its linear projection to a lower dimension that captures most of the variance of the data. In doing so, the resultant lower dimensional dataset becomes easier to explore and visualize, thus facilitating interpretability. The reduction of dimension is accomplished by transforming original features to a new set of features which are referred to as the Principal Components (PCs). The PCs are uncorrelated and ordered. Typically, most of the variation present in all of the original features is now retained in their first few PCs. Each PC is a linear combination of the original features. Higher loading (weight) reflects a larger contribution of a specific morphological feature to this component to the overall variation of data. Algebraically, PCs are obtained by projecting the original data into the direction of its highest variance.

Before applying PCA our dataset consisted of 15 parameters. We used Pearson correlation coefficient on the dataset to check which morphological features are linearly correlated. A correlation between pairs of features is computed and if two pairs are highly correlated > 0.8, one of the features is randomly removed. Among the 15 parameters, two pairs of features exhibited significant overlap: Euclidean distance and path distance (*r* > 0.8), and number of bifurcations and number of branches (*r* > 0.9). Path distance and number of branches were retained. After data preprocessing we kept 13 morphological features: branch contraction, convex hull, partition asymmetry, fractal dimension, number of neuronal stems, branch order, remote bifurcation amplitude, branch length, path distance, number of bifurcations, neuronal width, neuronal depth, neuronal height. Univariate analysis, including histograms, skewness analysis and boxplots was used to detect and remove outliers from the data (2%). Subsequently, we used the reduced dataset of 13 parameters to conduct both PCA and the Hierarchical clustering of the sample neurons using Euclidean distances as the distance measure and Ward’s method as the linkage rule. This clustering was accomplished to obtain clusters of basal dendritic trees that share similar morphological features. To visualize data from the cluster analysis, we used a TreeAndLeaf method as an alternative to the traditional dendrogram representation (Kume et al., 2020).

### Validation

In order to cross-validate the PCA model used to reduce the dimensionality of data, the original raw data set was randomly split into scaled training and test subsets in a 70/30 ratio, respectively. Two validation methods were subsequently used, one without PCA and another with PCA. For the first approach, we used the training dataset to train a Support Vector Machine (SVM) classifier using the age as a classification class and obtained a test accuracy score of 87%. For the second approach, we applied PCA on the training dataset. We then used the first n components (*n* = 6,6,7,9,4 in V1, V2, V4, TEO, and TE, respectively) that contributed to 95% of the variance to train an SVM classifier and obtained an accuracy test score of 84%. In order to obtain an estimate of the model performance on unknown data we used K-Fold Cross Validation estimator. This algorithm works by splitting the data into k folds/subsets where each k fold is used as a test/validation set and the k-1 remaining folds are used for training/fitting. This process is repeated k times and a mean evaluation score is then obtained. K fold validation reduces bias since every k-1 fold is used for training. It also reduces variance as every k fold of the data is also used for validation. We used a 10-fold validation method which resulted in an average 10-fold performance score of 83% using the first approach (raw data + SVM + k-fold), and a performance score of 82% using the second approach (PCA + SVM + k-fold).

## Results

The objective of the present work was to quantitatively characterize structural differences among pyramidal cells in visual cortical areas V1, V2, V4, TEO, and TE throughout development in an effort to reveal which morphological features change with age. Secondarily, we wished to determine if developmental changes in key morphological features occur simultaneously or hierarchically in multiple visual cortical areas. This was accomplished by using PCA and Hierarchical clustering analysis. PCA was used to identify underlying morphological features that differ among the different age groups, and thus revealed how much of the variability can be accounted for by specific features.

### Developmental Changes in Basal Dendritic Field Area

**FIGURE 1:**
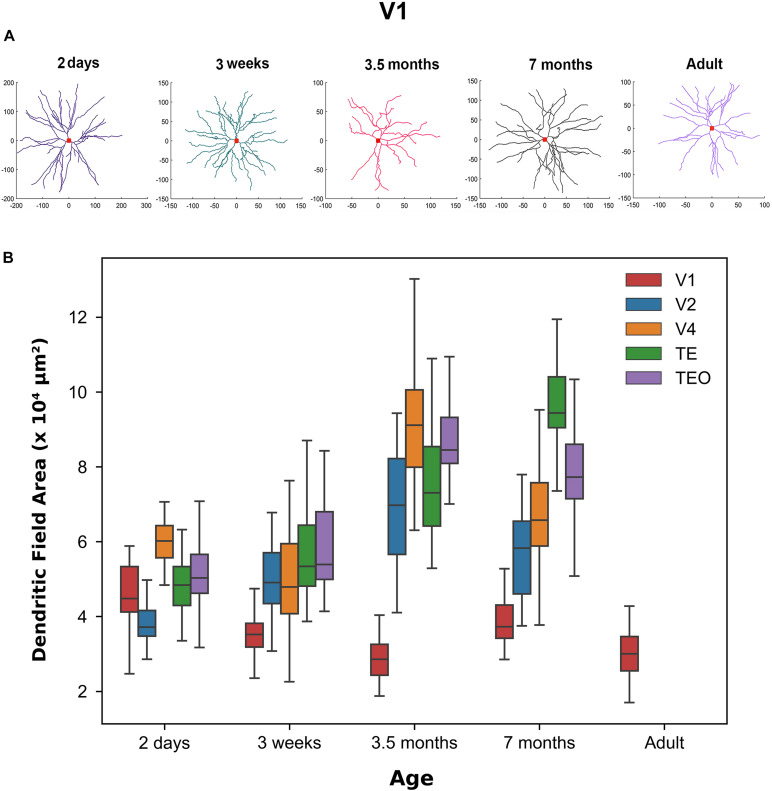
Differences in morphological features across age groups. **(A)** Representative reconstructions of the basal dendritic tree of layer III pyramidal cells in V1 at different ages. Red square at the center of the reconstruction represents the soma. **(B)** Box plot of dendritic field area in different visual cortical areas throughout development. Horizontal bars indicate median values and whiskers reflect the minimum and maximum values. Modified from Elston et al. (2010). Permission to adapt Figure 3 from Elston et al. (2010) was obtained from the licensed content publisher Oxford University Press with license number 5067250856403.

Figure 1A shows representative neuronal reconstructions of the basal dendritic tree of layer III pyramidal cells in V1 at different ages considered in this paper. The dendritic field surface area appears larger in newborns and progressively decreases with age, as can be seen by the different scales. Therefore, we were interested in quantitatively measuring changes in the dendritic field size throughout development as prior reports (Elston et al., 2010) have noted a decrease in this morphological measure into adulthood. Using the digital reconstructions in the SWC files we computed the size of the dendritic field (convex hull) as the area contained within a polygon joining the outer distal points of dendritic processes in 2 dimensions (Elston and Rosa, 1997). We plotted the dendritic field area for all visual cortical areas throughout development (Figure 1B). Indeed, in V1 this measure appears to progressively decrease from 2 days to 3.5 months of age, followed by a subsequent increase from 3.5 to 7 months of age. Dendritic field surface area decreases again from 7 months to adulthood. Therefore, by 3.5 months of age the surface area of the basal dendritic tree appears adultlike (median value at 3.5 months = 28,547 μm^2^ while in adult = 30,011 μm^2^). Conversely, cells in TEO and TE become progressively bigger from 2 days to 7 months of age (TE, median value at 2 days = 48,376 μm^2^ while at 7 months = 94,416 μm^2^, TEO, median value at 2 days = 50,300 μm^2^ while at 7 months = 77,270 μm^2^). In V4, cells appear to increase in size from 3 weeks to 3.5 months of age, and then decrease substantially to 7 months of age (median at 7 months = 65,770 μm^2^). Given that we do not have adult data for V2, V4, TEO, and TE, we cannot exclude further changes in dendritic field surface area beyond 7 months of age. Nevertheless, these data suggest that developmental changes in the size of cells in all areas examined is characterized by different trajectories. Moreover, cells in V1 appear to decrease in size from 2 days to adulthood, and are substantially smaller than cells in other areas at all ages examined except at 2 days of age.

**FIGURE 2:**
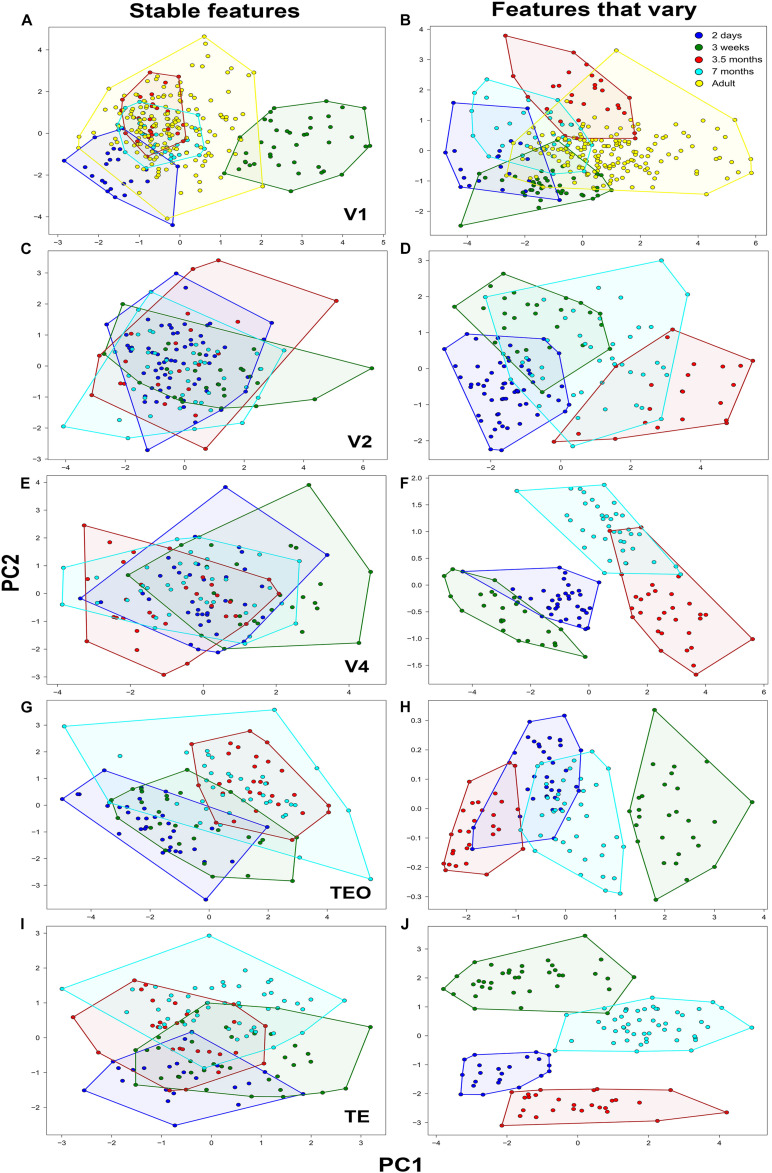
Principal component analysis of basal dendritic trees based on stable features and features that vary (*N* = 782). Plot of the first two PCs, Principal component 1 (PC1) and Principal component 2 (PC2) in **(A,B)** V1, **(C,D)** V2, **(E,F)** V4, **(G,H)** TEO, and **(I,J)** TE. Neurons comprising a cluster in each age group are found in neighboring positions and are delineated with a polygon.

**FIGURE 3:**
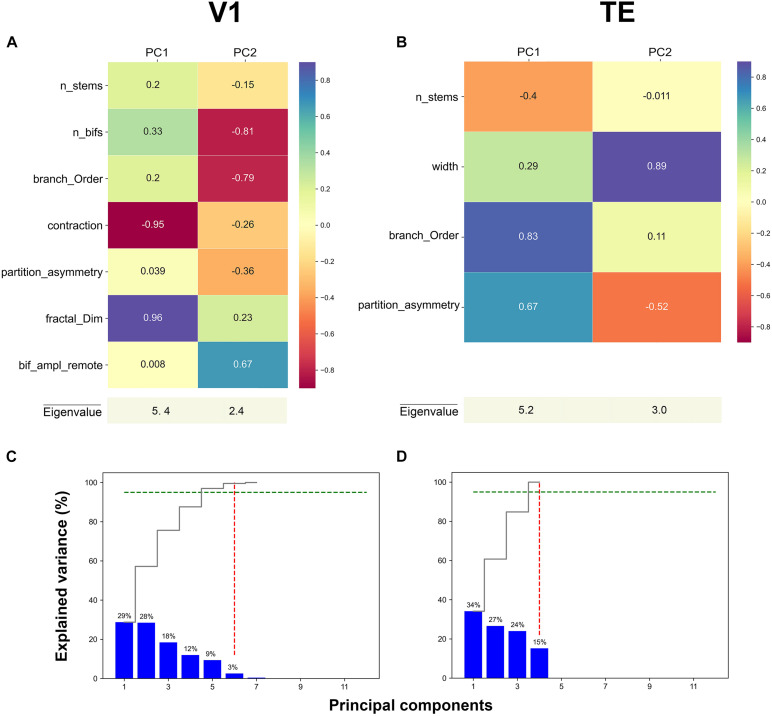
First two principal component loadings of basal dendritic tree measurements in **(A)** V1, and **(B)** TE. The stable parameters and their respective values in each Principal component axis (PC1 and PC2). A higher value means that the measurement has a larger contribution to the data variance on the axis. Cumulative and individual explained variance in the PCA in **(C)** V1 and **(D)** TE. The dashed green line marks the 95% explained variance and the dashed red line corresponds to the component number. Six and four components explain 95% of the variance in the data (intersection of dashed green and red lines).

### Principal Component Analysis of Dendritic Morphology

PCA allowed us to determine which aspects of neuronal morphology contribute most in differentiating pyramidal cells at each age during development. This method aims to maximize the variance present in the data in its initial dimensions. By restricting our analysis to the first two PCs which contain at least 50% of the variance in the data, we were able to achieve separation of age groups based on morphological features that contributed the most to PC1 and PC2. However, separation of groups does not necessitate maturation of particular morphological features since it does not reveal which features stabilize during development and which continue to change. Several scenarios can result in separation of age groups in the PC plot. For instance, it is conceivable that a particular morphological feature is characterized by a sinusoidal curve with values increasing and decreasing throughout development. Such a feature will exhibit a large variation and therefore will be prominent in the initial PCs. Alternatively, a feature may increase or decrease monotonically without stabilizing. Since we are also interested in revealing which particular morphological features stabilize during development and which ones continue to mature, we applied PCA in two different ways to distinguish these possibilities. Specifically, PCA was applied separately on stable features, and on features that varied. Separate PCA analysis allowed us to reveal developmental changes in the general morphology to determine which features stabilize and thus contribute significantly to the maturation of pyramidal cells. We accomplished this by first measuring developmental changes in each morphological feature to determine if the stabilization of a particular feature is achieved by 3.5 months of age. We generated normalized histograms of each morphological feature at 3.5 and 7 months of age in all visual cortical areas (7 months and adult in V1), and used the difference between them as a measure of a distance. If the distance between the histograms of the two age groups was small, the respective morphological feature was deemed stable. After careful inspection of the obtained histograms, a criterion cutoff distance of 1 was established to ensure stabilization of a feature. If the distance between histograms is less than one, the feature was deemed stable. However, if this value was greater or equal to 1, this was indicative of features that continue to mature (Table 2). The smaller the difference between the overlapping histograms reflects greater stability in a particular feature. We thus wanted to compare how PCA would separate the age groups when only stable features were used in the analysis versus features that vary.

**TABLE 2:** Morphological features that were used in PCA for all visual cortical areas.

	**V1**	**V2**	**V4**	**TEO**	**TE**
n_stems	0.26	0.16	0.38	0.44	0.49
branch_Order	0.39	0.36	0.60	0.28	0.79
n_bifs	0.85	0.62	0.77	0.66	1.20
bif_ampl_remote	0.57	0.58	0.88	0.76	1.01
partition_asymmetry	0.74	1.18	0.69	0.66	0.61
Length	1.30	0.71	0.82	0.75	1.34
Width	1.01	1.04	1.19	0.77	0.93
pathDistance	1.02	1.03	0.84	0.58	1.55
Height	1.30	1.17	1.10	0.97	1.40
Depth	1.03	0.48	1.37	0.99	1.51
Contraction	0.60	1.36	1.68	1.90	1.93
fractal_Dim	0.62	1.53	1.31	1.88	2.00
convex_hull	1.28	1.03	1.16	0.80	1.28

*Values that are less than 1 reflect stable features while values greater or equal to 1 reflect features that vary.*

The distribution of the descriptors of all pyramidal cells in the reduced 2D space which was obtained to facilitate analysis and visualization of the data is shown in Figure 2. This was accomplished by projecting the data to the main two PCs using stable features and features that vary in all visual cortical areas. Individual clusters comprising cells in the same age group are delineated with a polygon obtained from the cluster analysis to facilitate visualization. Most neurons from the same age group were located in neighboring positions resulting in clearly defined clusters corresponding to the age groups.

When PCA was applied on stable morphological features, V1 pyramidal neurons in the 3-week old age group constituted a clearly identifiable largely non-overlapping cluster (Figure 2A), while neurons in the 2-day old group showed moderate overlap with the adult group. However, the clusters comprising neurons in the 3.5- and 7-month old groups overlapped completely with neurons in the adult group. It should be noted that this is not purely a consequence of the selection of the stable features, as normalized histograms of a distance at most 1 can still differ considerably. Since those differences are not visible in the first two PCs, they are attributed to lower variance directions that can be considered noise in the data. The first component (PC1) differentiated the 2 day old and 3 week old groups from the 3.5, 7 month, and adult groups explaining 29% of the variability. Fractal dimension and contraction were the most important features in differentiating the age groups in PC1, while in PC2 the features that differentiated the age groups were number of bifurcations and branch order. Likewise, the second component (PC2) differentiated primarily the 2-day old from the remaining age groups and explained 28% of the variability. Here, the number of bifurcations and branch order contributed the most to the differentiation of age groups, suggesting that by 3 weeks of age these morphological features are comparable to that found in the adult. When PCA was applied on only those features that vary (i.e., did not stabilize), there was little differentiation in terms of PC1 as evidenced by the overlap of the age groups (convex hull explained 67% of the variation in PC1) (Figure 2B). However, some of the data comprising the adult cluster was clearly separated from the other age groups in PC1. This suggests that convex hull continues to change beyond 3.5 months of age since the cluster comprising this age group only shows moderate overlap with the adult cluster. There was better differentiation in the second component (PC2) between the age groups whereby depth contributed most in this case and explained 16% of the variability, again suggesting that this feature continues to mature during development.

In V2, when stable features were used in the PCA analysis (Figure 2C) neurons in all age groups were completely overlapped with little differentiation among groups both in PC1 and in PC2. Number of bifurcations and branch length contributed the most to the variance observed in PC1 and accounted for 40% of the variability, while number of stems and bifurcation amplitude local contributed the most to the observed variance in PC2 explaining 20% of the variability. PCA analysis based on features that vary resulted in moderately distinct clusters with some overlap between the age groups (Figure 2D). In general, the separation of clusters was mostly in terms of PC1, while there was minimal differentiation in terms of PC2. The first component (PC1) differentiated the 2 day old and 3 week old data from the 3.5 month old data, with convex hull contributing the most to the variance and accounting for 60% of the variability. Likewise, the second component mainly differentiated the 2 day old and 3 week old groups, with fractal dimension and contraction contributing the most to the variance and accounting for 19% of the variability. Similarly, in V4 PCA analysis based on stable features resulted in largely overlapping clusters of neurons in the different age groups (Figure 2E). Branch length and number of bifurcations contributed the most to the variance observed in PC1 and accounted for 41% of the variability, while branch order and partition asymmetry accounted for most of the observed variance in PC2 explaining 20% of the variability. When PCA was applied on only those features that vary, distinct clusters of neurons in each group were observed in terms of PC1 as (height and convex hull explained 76% of the variation in PC1) (Figure 2F). There was also clear differentiation in PC2 between the 2 day old, 3 week old, and 7 month old age groups.

In TEO, analysis of neurons using PCA on stable features resulted in distinct clusters in the 2-day old and 3.5 month old groups, while the 3 week old and 7 month old group exhibited moderate overlap (Figure 2G). Differentiation among neurons in the different age groups in TEO was mainly in PC1 with little separation of clusters in terms of PC2. Convex hull and path distance contributed the most to the differentiation of neurons in PC1 and accounted for 40% of the variability, while the number of bifurcations and branch order accounted for most of the observed variance in PC2 explaining 15% of the variability. When PCA was applied on features that vary, differentiation was mostly observed in terms of PC1 as evidenced by the distinct clusters comprising the age groups (contraction and fractal dimension explained 99% of the variation in PC1) (Figure 2H). There was no differentiation in terms of PC2 whereby contraction and fractal dimension accounted for 1% of the variability. Lastly, PCA applied on stable features in TE resulted in distinct clusters comprising the 2 day old and 7 month old data while data from the 3 week old and 3.5 month old groups was mostly overlapping (Figure 2I). There was little differentiation in terms of PC1 whereby branch order and partition asymmetry accounted for 34% of the variability. Differentiation was mostly observed in terms of PC2 width and partition asymmetry contributed the most to the variance and explained 34% of the variability in the data. When PCA was applied on features that vary, differentiation was mostly observed in terms of PC2 as evidenced by the distinct clusters comprising the age groups (fractal dimension and contraction explained 48% of the variability in PC2) (Figure 2J). There was moderate separation observed in PC1 whereby length and height explained 29% of the variability in the data.

The PCA weights given by the respective eigenvector components of the two principal main axes for representative areas V1, and TE are shown in Figures 3A,B (for other areas see Supplementary Figure 1). These values were obtained when PCA was applied on only stable features. In the first axis for V1, the morphological features that contributed the most to PC1 were: contraction (–0.95) and fractal dimension (0.96) (Figure 3A, **magenta** and **purple** colors). Likewise, in the first axis for area TE, the morphological features that significantly contributed the most to PC1 were: branch order (0.83) and partition asymmetry (0.67) (Figure 3B, **purple** and **turquoise** colors). Therefore, it seems that morphological features associated with topological aspects of the dendritic tree such as branch contraction, branch order, fractal dimension, and partition asymmetry differentiated the age groups which suggests that these features continue to mature during development. Conversely, the morphological feature that contributed the most to PC2 in V1 was width (0.89), while number of bifurcations (–0.81) and branch order (–0.79) were the features that contributed the most in area TE (Figures 3A,B, **magenta** and **purple** colors). Figures 3C,D show the variance explained by each of the principal axes in both V1 and TE. This was computed using the eigenvalues: higher values reflect a higher contribution. Each plot shows the eigenvalues that were converted into percentages and presented in a cumulative sequence of bars, emphasizing the cumulative contribution of each PC for the data variability. The first two eigenvalues used in the PCA plots explained 57% of the variance in area V1, and 61% of the variance in TE.

### Cluster Analysis of Neuronal Morphology Across Development in TE

**FIGURE 4:**
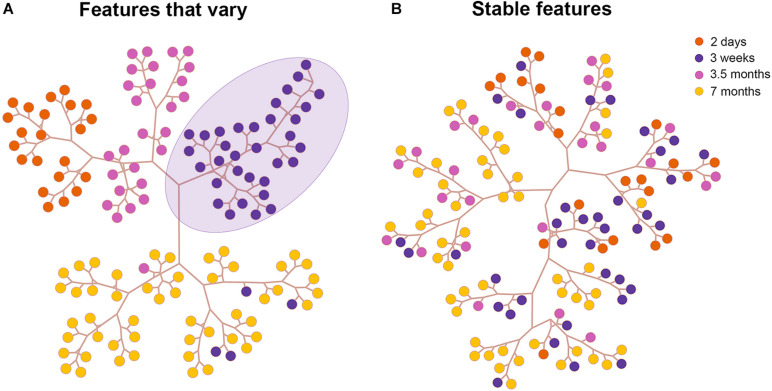
Results of the cluster analysis in area TE. Tree and leaf representation of basal dendritic trees in all age groups in TE based on features that vary **(A)** and on features that are stable **(B)**. Each colored circle in the tree depicts a neuron from an age group. The purple circles delineated by a lavender ellipse are clustered together on a branch since they are morphologically similar.

To validate our PCA results we next performed Hierarchical cluster analysis via Ward’s method (Ward, 1963) and Euclidean distance using the first n components that contributed to 95% of the variance in the reduced dataset in all visual cortical areas. Representative clustering trees of neurons in TE based on stable features (Figure 4A) and features that vary (Figure 4B) are shown (for other areas see Supplementary Figures 2, 3). This is a graphical representation comprising the different clusters acquired based on linkage distances. Each colored circle in the tree depicts a neuron from an age group. Neurons clustered together on a branch (3 week old purple circles delineated by lavender ellipse in Figure 4A) share greater morphological similarity than neurons on different branches. Indeed, the cluster analysis based on features that vary revealed that most of the pyramidal cells in each age group were largely clustered together on a branch. For instance, neurons in the 2 day old group (orange circles) were found on a single branch suggesting similarity in morphology. Likewise, pyramidal cells in the 3 week old group were clustered together and found on their own branch (purple circles). Cluster analysis which included only stable features resulted in pyramidal cells that were largely intermingled among the age groups (Figure 4B). These findings mirror our PCA results and suggest that since these features stabilize during development, they are morphologically similar in all the age groups. This is reflected in the clustering tree by the large degree of intermingling of neurons from the different age groups.

## Discussion

The goal of the present work was to examine morphological differences of layer III pyramidal cells in cortical areas of the ventral visual pathway (V1, V2, V4, TEO, and TE) of the macaque monkey throughout development in an effort to establish which aspects of neuronal morphology mature early in development and which features require a protracted period of maturation. Secondarily, we wished to determine if developmental changes in morphological features occur simultaneously or hierarchically in multiple visual cortical areas. The publicly available database of digital reconstructions http://neuromorpho.org/ enables data exploration and reanalysis of neuronal morphology to further our understanding of brain structure and function in the adult and developing brain. This was the primary focus of this work. We separately performed PCA on stable features and features that vary throughout development to tease apart the contribution of each set of features to the differentiation of age groups in the PC plots. Our findings reveal that topological aspects such as branch order, fractal dimension, and contraction of the dendritic tree of layer III pyramidal cells mature early in development while morphological features related to the size of the dendritic tree continue to mature. This is particularly true for areas TEO and TE in which the majority of morphological features continued to mature during development. Moreover, the temporal sequence of developmental changes in key morphological features appeared to differ across visual cortical areas.

Changes in dendritic field area of layer III pyramidal cells in each visual cortical area throughout development is characterized by different developmental trajectory (Figure 1A). For instance, the dendritic field area of layer III pyramidal cells in V1 resembles the adult state by 3.5 months of age (Elston et al., 2010). Conversely, this measure increases throughout development in areas TEO and TE. Given that we do not have adult data for V2, V4, TEO, and TE, we cannot exclude further changes in dendritic field surface area beyond 7 months of age. Differences in the developmental trajectories of the dendritic field surface area of cells among the cortical areas is not mirrored by timing of peak spine density shown by Elston et al. (2010). The authors revealed that peak spine density occurred in all cortical areas at 3.5 months of age. Nevertheless, although peak spine density was coincident in all cortical areas at 3.5 month of age, the growth and pruning of spines appears to differ among the areas. In V1, V2, and V4, there was a decrease in spine number within the dendritic trees of layer III pyramidal cells from 2 days to adulthood, while more spines were grown in TEO and TE from 2 days to 3.5 months of age, which are then pruned as cells mature to adulthood. Our results also show that this population of V1 pyramidal cells in the 2-day old and 3-week old age groups is characteristically different from those found in the adult as seen in the PCA plot that was applied on stable features (Figure 2A). This is in stark contrast to the complete overlap of clusters comprising the 3.5 and 7 month old age groups with that of the adult. Differentiation of the 2 day old and 3 week old groups from the 3.5 month, 7 month and the adult group in PC1 was largely based on fractal dimension and contraction. This suggests that fractal dimension and contraction continue to mature from 2 days to 3 weeks of age, as evidenced by the non-overlapping clusters. However, these morphological features are mostly mature by 3.5 months of age since this cluster was completely overlapped with the adult data.

It’s worth mentioning that when PCA is applied to features that are stable we expect to observe a large degree of overlap between the age groups, and therefore any separation of clusters comprising the different age groups reflects variations between the younger and older animals. In other words, since fractal dimension and contraction were among the stable features used in the PC plot in Figure 2A, separation of the 2 day old and 3 week old groups suggests early maturation of these features, ultimately stabilizing by 3.5 months of age. Conversely, when PCA is applied on features that vary we expect to observe distinct clusters of cells with some degree of overlap since these features vary throughout development and do not stabilize. Fractal analysis is a method to quantify dendritic branching patterns (i.e., proxy for branching complexity), and is more sensitive in revealing potential differences than classical Sholl analysis. For instance, fractal analysis revealed a systematic increase in dendritic complexity in the occipitotemporal pathway where this measure was lowest in V1 and highest in TEO/TE (Elston and Jelinek, 2001). Moreover, it has been shown that fractal dimension of layer V basal dendritic tree of pyramidal cells in V1, TE, and area 12 is comparable at birth, but branching complexity increases in area TE in the adult (Oga et al., 2017). The authors also report variations in fractal dimension (up and down) throughout development in all areas. Conversely, changes we observe in the present study in basal dendritic arbors of pyramidal cells in layer III of V1 and TE appear to be different than that found in layer V of these visual cortical areas. Pyramidal cells in layer V of V1 retract basal dendritic arbors, whereas those in area TE remained constant in size resulting in a dendritic field area that remains unchanged throughout development. Thus, these results suggest that temporal profiles of morphological features are not only area specific but also vary according to cortical layer.

PCA analysis based on stable features in V4 resulted in largely overlapping clusters of neurons in the different age groups (Figure 2E). Branch length and number of bifurcations contributed the most to the variance observed in PC1. The fact that the 3 week old cluster and the 3.5 month old cluster are on opposite ends of the plot suggests that there is maturation of branch length and number of bifurcations early in development that ultimately stabilize by 3.5 months of age as evidenced by the overlap of this cluster with the 7 month old cluster. However, further changes cannot be excluded since adult data is not available. When PCA was applied on stable features in area TE, differentiation was mostly observed in terms of PC2 whereby width and partition asymmetry contributed the most to the variance in the data. Since there was minimal overlap between the 2 day old and 7 month old age groups, this suggests that width and partition asymmetry of pyramidal cell dendritic trees in TE continue to mature throughout development. It is likely that further changes ensue beyond 7 months of age. However, we cannot determine that since adult data in this area was not available.

Revealing the timing of cortical circuit maturation is important as it reflects functional maturity. However, a precise definition of neural maturation is elusive and challenging, as metrics of maturation can vary widely and range from cellular morphology to biochemical expression profiles, to electrophysiological characteristics (Guillery, 2005; Bourne and Rosa, 2006; Elston and Fujita, 2014). A sensible approach to defining maturity would include a combination of parameters at different developmental stages (Guillery, 2005). Therefore, to accurately map developmental trajectories and define maturity, research aimed at revealing which morphological parameters are crucial for adult cortical function is needed. This in turn will illuminate neurobiological mechanisms involved in brain maturation and cognition (Chomiak and Hu, 2017).

The developmental refinement of pyramidal cells among different cortical areas exhibits distinct developmental profiles (Elston et al., 2009, 2010, 2011, Elston and Fujita, 2014). For example, layer III pyramidal cells in inferotemporal cortex (IT) increase the size of their dendritic trees after the peak in synaptogenesis into adulthood (Elston et al., 2010). Likewise, there is an increase in the size and complexity of the dendritic tree of layer III pyramidal cells in the anterior ventral inferotemporal cortex (IT) (Elston et al., 2011). Conversely, dendritic trees of layer III pyramidal cells in primary visual cortex (V1) of the macaque monkey decrease in size from birth into adulthood (Boothe et al., 1979; Elston et al., 2010). Developmental growth profiles of pyramidal cells also appear to be layer specific. Indeed, quantitative measurements of the dendritic field size of layer III cells in V1 decrease throughout development and are essentially adultlike by 3.5 months of age (Elston et al., 2010), whereas in layer V of V1 there is a progressive decline in this measure into adulthood (Oga et al., 2017). Given that pyramidal cells in different cortical areas have specialized physiological properties, the observed variability in the time course of pyramidal cell development may be governed by the specific functional role of a cortical area in the adult brain. For example, layer III pyramidal cells in anterior ventral IT of the adult macaque mediate visual recognition (Mishkin and Murray, 1994), while layer III pyramidal cells in area MT respond to the direction of visual motion (Dubner and Zeki, 1971; Rodman and Albright, 1989).

What is clear is that many aspects of cortical architecture mature in a hierarchical manner such that lower order areas develop ahead of higher order cortical areas (Condé et al., 1996; Bourne and Rosa, 2006; Petanjek et al., 2008; Bianchi et al., 2013; Elston and Fujita, 2014). This mechanism of sequential maturation makes sense since it allows basic functions subserved by lower order cortical areas to stabilize which are necessary for the establishment of higher order functions. Delayed maturation of neuronal response properties and complex functions mediated by higher order cortical areas has been well documented. For instance, in infant monkeys, sensitivity to luminance-defined form matures later in development relative to both texture- and contrast-defined forms (El-Shamayleh et al., 2010). Evidence suggests that area V4 and higher areas are important in mediating texture segmentation (De Weerd et al., 1996; Huxlin et al., 2000). The late maturation in maximum size and maximum branching complexity in the dendritic trees of neurons in V4 which occurs at 3.5 months of age, could underlie the late development of sensitivity to luminance-defined form. Other visual functions that require a protracted period of development include; the ability to detect global structure patterns which does not occur before 12 weeks of age (Kiorpes and Movshon, 2003), and the ability to identify extended contours which is detected 20 weeks of age (Kiorpes and Bassin, 2003). Similarly, in humans, the ability to perform a contour integration task (Kovács et al., 1999) requires several years to develop and is not detected before the age of 3 years. These complex tasks which require extensive integration of visual features across space, are late to develop presumably because they depend on the maturation of higher order visual areas.

There is also evidence supporting delayed maturation of neuronal response properties in higher order cortical areas in monkeys. For instance, receptive fields of neurons in V2 mature later than those in V1 (Zhang et al., 2005). These physiological findings are in line with anatomical results we and others (Elston and Fujita, 2014) have shown in that the dendritic trees of pyramidal cells in V2 continue to grow from 2 D to 31/2 months of age while those in V1 become smaller during this time. This is evidenced by our PCA results when analysis was carried out on morphological features that vary in V2. Differentiation of age groups was mostly in the first component (PC1), separating the 2 days old and 3 weeks old data from the 3.5 months old data, with convex hull contributing the most to the variability in the data. This suggests that the size of the dendritic tree layer III pyramidal cells in V2 increases from 2 days to 3.5 months of age.

Separate PCA analysis conducted in the present work provides unique information by revealing key morphological features that significantly contribute to separation of cells among the age groups, effectively revealing which features continue to mature throughout development. Importantly, our results on the development of pyramidal cell structure in V1 is consistent with aspects of physiological and behavioral development (for reviews, see Elston and Fujita, 2014; Luebke, 2017). For instance, while orientation selectivity is present at birth in macaques, direction preference develops to adult levels over the first 4 weeks of postnatal life (Hatta et al., 1998). Similarly, spatial contrast sensitivity develops rapidly during the first 10–20 weeks of life, but full maturation to adult levels continues until the end of the first postnatal year (Boothe et al., 1988). Additionally, the receptive field size of infant macaque V1 neurons is substantially larger than that of the adult (Movshon et al., 2000; Zhang et al., 2005), and the spatial resolution of V1 cells increases from early in development into adulthood (Movshon et al., 2000). The improvement throughout development in spatial contrast sensitivity, and spatial acuity, coupled with a reduction in receptive field size could underlie the changes we and others (Elston et al., 2010) have observed in the size of the dendritic trees. This is because pyramidal neurons that have smaller dendritic fields may integrate inputs over a smaller region of cortex than larger cells (Elston and Rosa, 2000). Therefore, as the size of the neuronal dendritic trees decrease during development, they could sample a progressively smaller portion of the visual map, and as a result will have smaller receptive fields. Smaller receptive fields in the adult translates to increased spatial resolution.

## Data Availability Statement

The publicly available datasets are analyzed in this study. This data can be found here: http://neuromorpho.org.

## Author Contributions

RK and AF conceived and designed the work. AF and PD designed the computational analysis and performed all the coding. RK, AF, and PD interpreted the results, and were responsible for writing and revising the manuscript. All authors critically reviewed and edited the manuscript.

## Conflict of Interest

The authors declare that the research was conducted in the absence of any commercial or financial relationships that could be construed as a potential conflict of interest.
