# Supplementary figures and images for: Developmental Changes in Pyramidal Cell Morphology in Multiple Visual Cortical Areas Using Cluster Analysis
Source: Front Comput Neurosci. 2021 May 31;15:667696. doi: 10.3389/fncom.2021.667696 (PMC8200563; doi:10.3389/fncom.2021.667696)

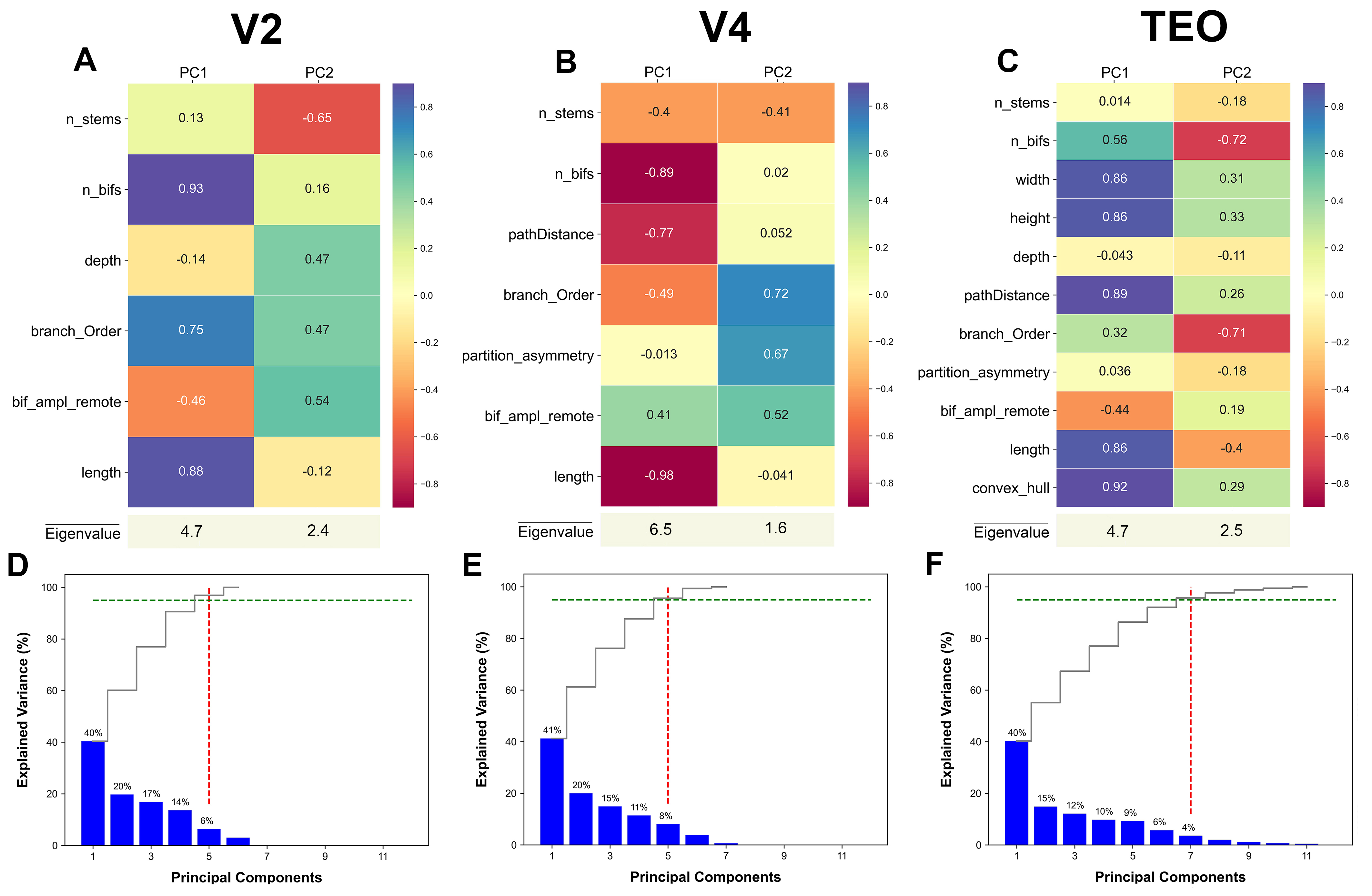

Supplement: Supplementary file 1 [file Image_1.JPEG]

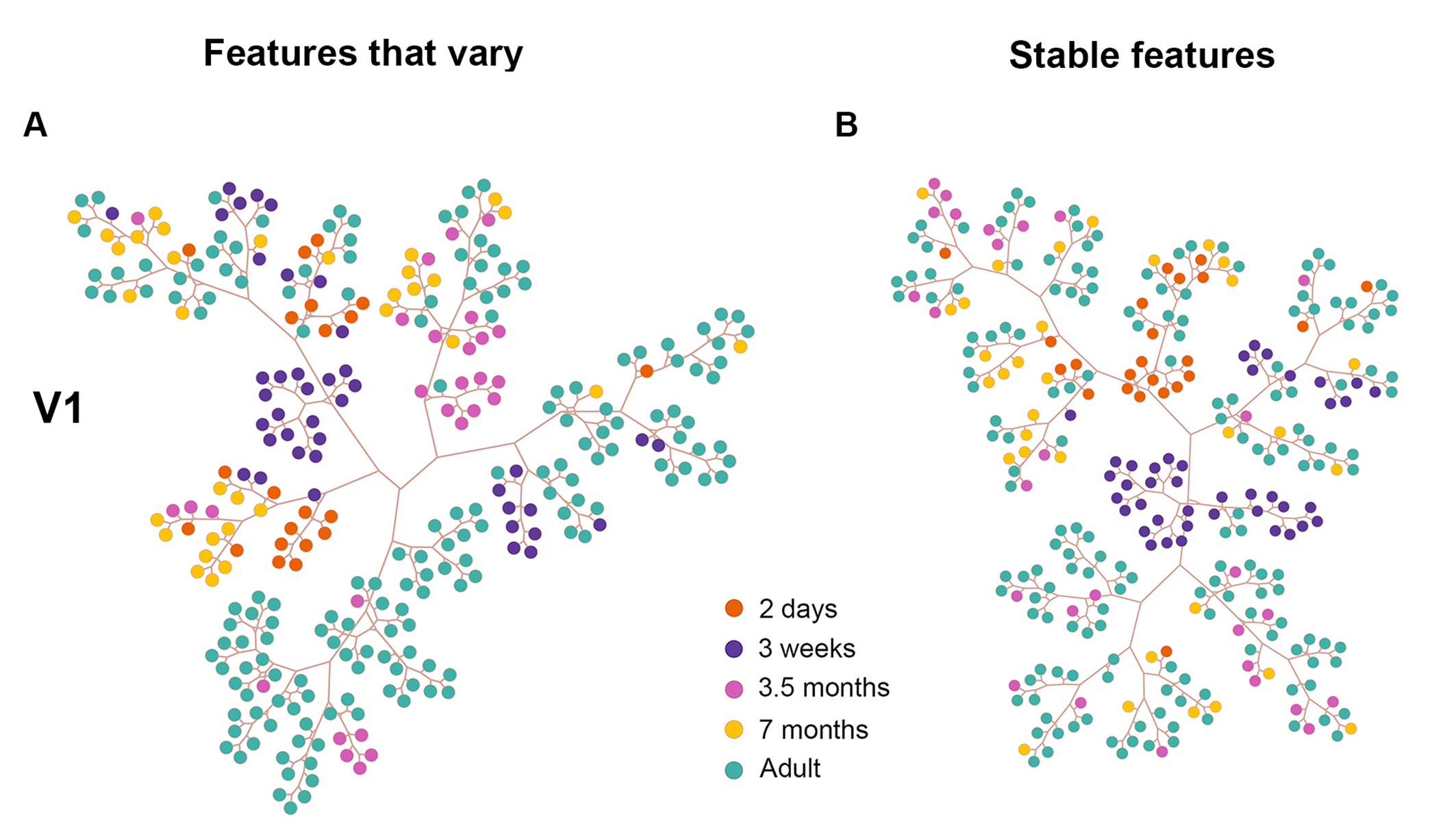

Supplement: Supplementary file 2 [file Image_2.JPEG]

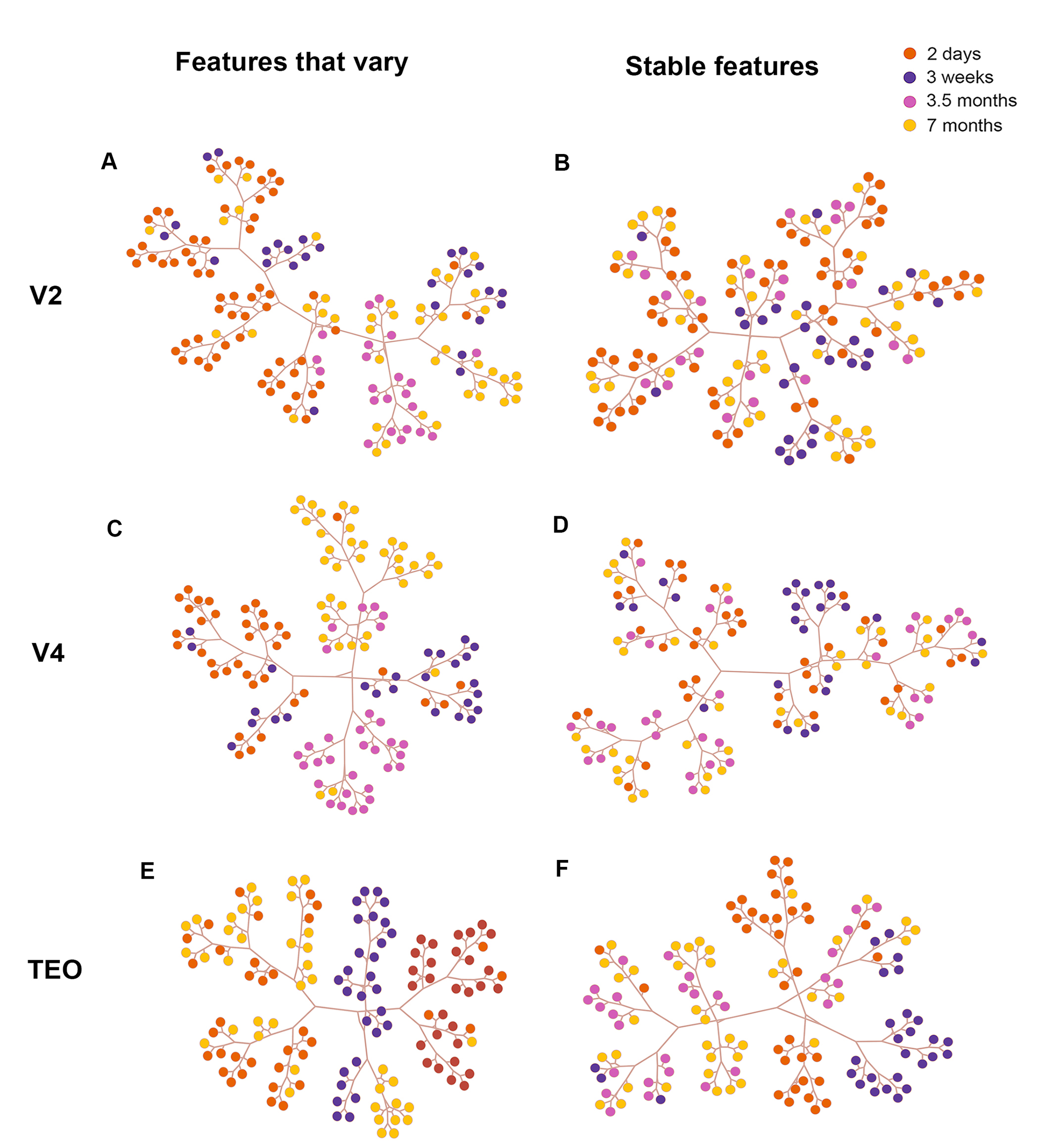

Supplement: Supplementary file 3 [file Image_3.JPEG]
